# Ambient air pollution as a time-varying covariate in the survival probability of childhood cancer patients in the upper Northern Thailand

**DOI:** 10.1371/journal.pone.0303182

**Published:** 2024-05-10

**Authors:** Lalita Sathitsamitphong, Imjai Chitapanarux, Pimwarat Srikummoon, Natthapat Thongsak, Nawapon Nakharutai, Salinee Thumronglaohapun, Titaporn Supasri, Phonpat Hemwan, Patrinee Traisathit

**Affiliations:** 1 Department of Pediatrics, Faculty of Medicine, Chiang Mai University, Chiang Mai, Thailand; 2 Northern Thai Research Group of Therapeutic Radiology and Oncology (NTRG-TRO), Divisions of Radiation Oncology, Faculty of Medicine, Chiang Mai University, Chiang Mai, Thailand; 3 Department of Statistics, Faculty of Science, Chiang Mai University, Chiang Mai, Thailand; 4 Atmospheric Research Unit of National Astronomical Research Institute of Thailand, Chiang Mai, Thailand; 5 Geo-Informatics and Space Technology Centre (Northern Region), Department of Geography, Faculty of Social Sciences, Chiang Mai University, Chiang Mai, Thailand; Kanazawa University, JAPAN

## Abstract

The objective of this study is to determine the possible association between exposure to air pollution and the risk of death from cancer during childhood in upper northern Thailand. Data were collected on children aged 0–15 years old diagnosed with cancer between January 2003 and December 2018 from the Chiang Mai Cancer Registry. Survival rates were determined by using Kaplan-Meier curves. Cox proportional hazard models were used to investigate associations of potential risk factors with the time-varying air pollution level on the risk of death. Of the 540 children with hematologic cancer, 199 died from any cause (overall mortality rate = 5.3 per 100 Person-Years of Follow-Up (PYFU); 95%CI = 4.6–6.0). Those aged less than one year old (adjusted hazard ratio [aHR] = 2.07; 95%CI = 1.25–3.45) or ten years old or more (aHR = 1.41; 95%CI = 1.04–1.91) at the time of diagnosis had a higher risk of death than those aged one to ten years old. Those diagnosed between 2003 and 2013 had an increased risk of death (aHR = 1.65; 95%CI = 1.13–2.42). Of the 499 children with solid tumors, 214 died from any cause (5.9 per 100 PYFU; 95%CI = 5.1–6.7). Only the cancer stage remained in the final model, with the metastatic cancer stage (HR = 2.26; 95%CI = 1.60–3.21) and the regional cancer stage (HR = 1.53; 95%CI = 1.07–2.19) both associated with an increased risk of death. No association was found between air pollution exposure and all-cause mortality for either type of cancer. A larger-scale analytical study might uncover such relationships.

## Introduction

Childhood cancer is of great concern worldwide. From 1990–2011, 3,574 Thai children, aged 0–19 years, were diagnosed with cancer: most of them were diagnosed with leukemia (34.8%), followed by brain and spinal neoplasms (12.3%) and lymphoma (11.2%) [1]. For all pediatric cancers, the age-standardized incidence rate (ASR) was 98.5 per million persons per year and the 5-year survival rate was 43.1% (95% confidence interval (CI): 41.1% to 45.2%). These data classified by age group, for children aged 0–4, 5–9, and 10–14 years old, the ASRs were 123.7, 73.1, and 82.2 per million persons per year, respectively, and the 5-year survival rates were 44.4% (40.7%, 48.4%), 40.7% (36.2%, 45.7%), and 44.3% (40.3%, 48.8%), respectively [1]. According to childhood cancer types, the high survival rate was for retinoblastoma, renal tumor, Hodgkin lymphoma and germ cell tumor compare to inferior outcomes in malignant bone tumor, acute myeloid leukemia and neuroblastoma [1, 2].

Air pollution comprising primary and secondary pollutants is associated with long-term harmful effects on organs and overall health. Air pollution causes the greatest number of deaths worldwide, with an estimated 6.7 million deaths in 2019 due to ambient air pollution (AAP) [3]. AAP is commonly defined as including particulate matter with an aerodynamic diameter < 2.5 μm (PM_2.5_) or < 10 μm (PM_10_), carbon monoxide (CO), ground-level ozone (O_3_), nitrogen dioxide (NO_2_), and sulfur dioxide (SO_2_) [4]. The recommended guidelines for AAP levels set by the World Health Organization (WHO) are PM_2.5_ ≤ 5 μg/m^3^ (annual mean), PM_10_ ≤ 15 μg/m^3^ (annual mean), O_3_ ≤ 100 μg/m^3^ (8-hour mean), NO_2_ ≤ 10 μg/m^3^ (annual mean), SO_2_ ≤ 40 μg/m^3^ (24-hour mean), and CO ≤ 4 μg/m^3^ (24-hour mean) [5]. Thailand’s National Ambient Air Quality Standards have recently been established as PM_2.5_ ≤ 15 μg/m^3^ (annual mean), PM_10_ ≤ 50 μg/m^3^ (annual mean), O_3_ ≤ 140 μg/m^3^ (8-hour mean), NO_2_ ≤ 57 μg/m^3^ (annual mean), SO_2_ ≤ 100 μg/m^3^ (annual mean) or ≤ 300 μg/m^3^ (24-hour mean), and CO ≤ 10,260 μg/m^3^ (8-hour mean) [6].

According to the findings from an annual report on the situation and management of air pollution and noise problems in Thailand in 2019, the northern region has air pollution levels that exceed the standard level for more than 73 days each year [7]. The main source of air pollution in northern Thailand is the burning of agricultural waste after harvesting from January–April [8, 9], during which the PM_2.5_ level is at its highest [10, 11]. The findings from an annual report on air pollution in northern Thailand produced in 2020 indicate that the maximum 24-hour average levels of PM_10_ and PM_2.5_ were 412 and 263 μg/m^3^, respectively, which are at levels that can seriously affect health [12]. A recent study on ambient airborne particulate matter in Thailand also indicates that the highest concentration occurs during the dry season: the northern area of Thailand has the highest PM0.1 concentration, followed by the central and southern areas [6]. In another study a year later [13], the authors also reported that the highest ambient particulate matter concentration occurs at night rather than during daylight.

In a study of the effects of ambient particulate matter and biomass burning on hospital visits in northern Thailand, Mueller, et al. [10] found a correlation between daily PM_10_ concentrations and outpatient visits for chronic lower respiratory disease (Incidence Rate Ratio (IRR) = 1.020; 95% CI = 1.012–1.028) and cerebrovascular disease (IRR = 1.020; 95% CI = 1.004–1.035). In Chiang Mai, Chiang Rai, and Nan provinces in northern Thailand, Leelasittikul, et al. [14] found an association between air pollution and the risk of pulmonary dysfunction in children and poor cardiovascular endurance in all age groups (children, adults, and the elderly). The findings from a previous study in iran also indicate that each 10 μg/m^3^ increase in PM_10_ and NO_2_ concentrations and 1 mg/m^3^ increase in CO concentration are significantly associated with 1.007-, 1.033-, and 1.094-fold higher risks of hospital admission for cardiovascular problems, respectively [15]. The findings from a meta-analysis indicate a 1.04-fold (95% CI = 1.02–1.06) higher mortality rate with an increase of 10 μg/m^3^ in the annual NO_2_ concentration [16]. Moreover, it has been reported that O_3_ exposure increases the risk of cardiovascular mortality while PM_10_ exposure is significantly associated with respiratory mortality [17].

The exposure to air pollution will deteriorate the health and treatment outcome of childhood cancer patients. Several studies in adult patients with cancer found a relationship between cancer mortality and air pollution exposure [18–23]. However, the risk of childhood cancer mortality with air pollution was not clearly elucidated. As previously published, it has been reported that mortality from childhood cancer is associated with the individual’s characteristics, such as gender (male patients have a higher risk than female ones) [24], age (younger patients have a higher risk than older ones) [25, 26], and socioeconomic status (SES) (a low SES increases the risk of death) [24, 25].

The aim of the present study is to evaluate the possible association between childhood exposure to air pollution and the risk of death in northern Thailand on data collected from the Chiang Mai Cancer Registry Maharaj Nakorn Chiang Mai Hospital (the center for cancer treatment and research in the northern region of Thailand) from 2003–2018 using survival analysis.

## Materials and methods

### Study population

**Fig 1:**
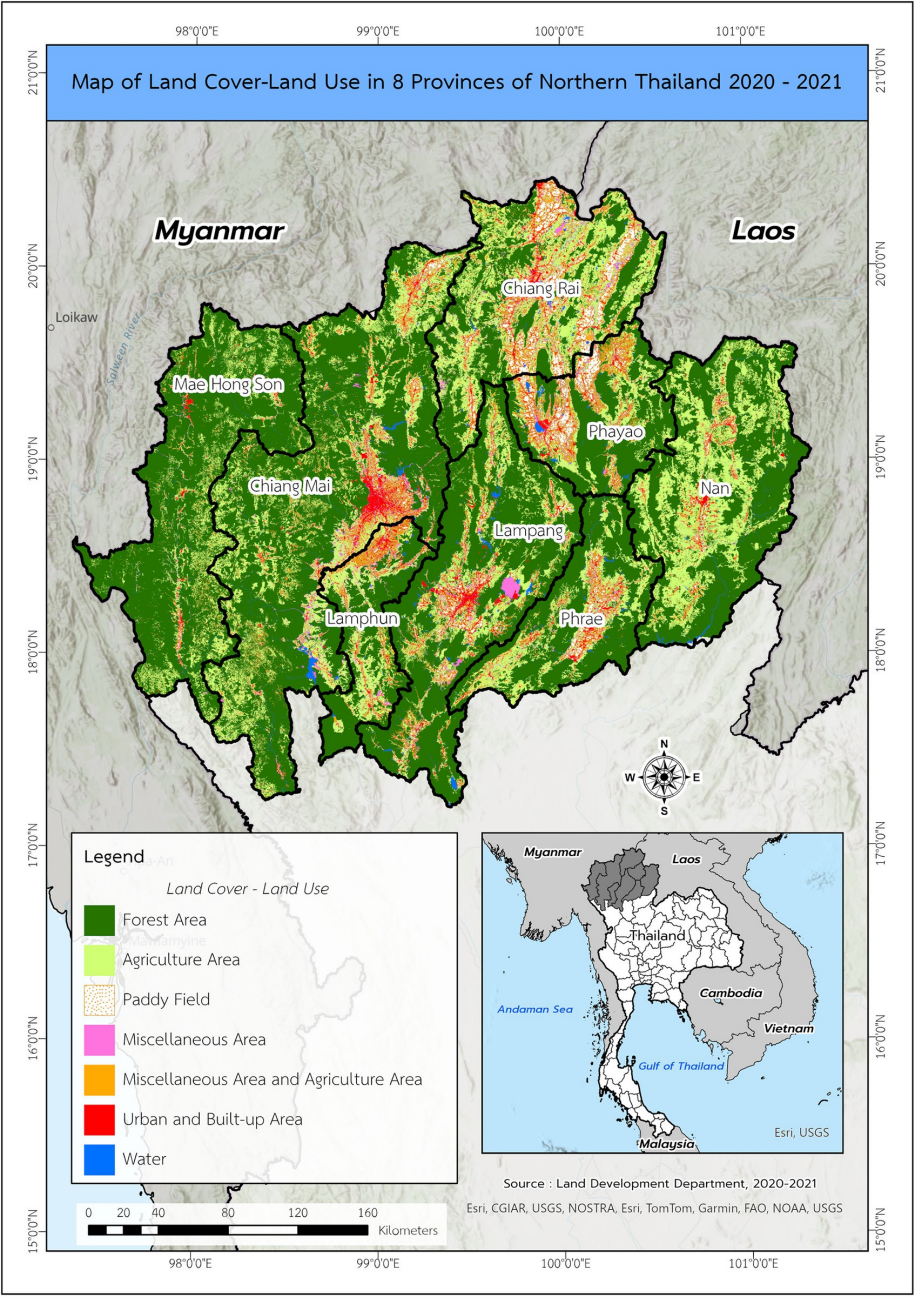
A map of the study area.

Data for the study population were retrieved from the Chiang Mai Cancer Registry for eight provinces in upper northern Thailand, including Chiang Mai, Chiang Rai, Nan, Phrae, Phayao, Lampang, Lamphun, and Mae Hong Son. A map of the study area can be found in Fig 1. These comprised clinical data on cancers in children aged 0–15 years old who were diagnosed from 1 January 2003 to 31 December 2018 and followed up to the end of 2020 (accessed 25 May 2021). Data for patients who died on the day of diagnosis were excluded from the study.

### Exposure assessment for time-updated variables

The Copernicus Atmosphere Monitoring Service (CAMS) provided the reanalysis datasets for the atmospheric composition obtained by modeling worldwide observation data with a spatial resolution of around 80 kilometers. The data are available as spectral coefficients with a triangular truncation of T255 (or approximately 80 km) or as a reduced Gaussian grid with a resolution of N128. CAMS provides datasets using an atmosphere model based on the laws of physics and chemistry [27, 28]. Currently, the available period for these datasets is from the beginning 2003 to the end of June 2022. They are suitable for conducting climatology computations, performing trend analysis, or comparative studies with other reanalysis models [29]. The reliability and validity reports on the CAMS Quality Assurance website for the data infer that the integrated system can be used to estimate the bias between observations and to separate high-quality data from inaccurate data. The atmosphere model provides estimations for areas with limited data coverage or for atmospheric contaminants for which no direct observations are available. Reanalysis is very convenient to ensure completeness of the data for each grid point around the world over a long period of time and in a similar format [30]. We accessed the three-hour AAP concentration levels of PM_2.5_ (kg/m^3^), PM_10_ (kg/m^3^), NO_2_ (kg/m^2^), SO_2_ (kg/m^2^), CO (kg/m^2^), and O_3_ (kg/m^2^) in upper northern Thailand for the study period (accessed on 27 November 2021). The concentration of each pollutant was calculated from the annual average concentrations separately for each district in the eight provinces in upper northern Thailand. We also assumed that the patient lived at their registered address. Thereby, we matched the annual average concentration of each air pollutant to the district where the patient lived for the year of cancer diagnosis and the follow-up years thereafter. The annual concentrations of pollutants were determined until either the death, was lost to follow-up, or was censored because of still being alive at the end of the study period.

### Baseline and follow-up data

Data for the cancer cases, including gender, age at diagnosis, and cancer characteristics (the type of cancer and cancer stage (SEER staging: localized, regional, or metastatic)) were obtained from the Chiang Mai Cancer Registry. The annual concentrations of air pollutants that the patients were exposed to from the year of diagnosis until the end of the study period were calculated from the air pollution data described in the previous subsection [29].

### Statistical analysis

This study was divided into two sections according to the groups of childhood cancer: hematologic malignancy (e.g., leukemia, lymphoma, or myelodysplastic syndrome) and solid tumors (e.g., central nervous system tumor, germ cell tumor, malignant bone tumor, etc.).

For the baseline characteristics, categorical variable values are presented as frequencies and percentages while continuous variable values are presented as medians and interquartile ranges (IQRs). The follow-up time was measured from the diagnosis date to whichever of the following occurred first: the date of death regardless of the cause, the last follow-up date, or being censored because of still being alive at the end of the study period.

To obtain the overall death rate for each variable, we divided the number of deaths by the total number of persons per year of follow-up (PYFU). Next, the confidence intervals (Cis) for the mortality rates were obtained after fitting the data to a Poisson distribution. Finally, we used Kaplan-Meier curves to produce survival rates and log-rank tests to ascertain significant differences between the survival probabilities of the various groups according to each variable.

We used Cox proportional hazard models to examine the relationships between survival time and specific risk factors. This method can predict hazard ratios representing the risk or probability of the time-related occurrence of all-cause mortality for potential risk factors as baseline or time-varying variables. The potential risk factors in this study include gender, age at diagnosis, cancer stage, calendar year of registration (grouped as 2003–2013 or 2014–2018: the periods before and after the establishment of the national protocol for the treatment of childhood cancers), and the time-updated concentrations of PM_2.5_, PM_10_, NO_2_, SO_2_, CO, and O_3_ during the study period [29]. For each continuous variable, we grouped the values by quartiles or separated them into two groups when appropriate or for the equivalent sample. The associated risk of death factors with a *p*-value < 0.25 in the univariable analysis were included in the multivariable analysis by using the backward elimination method. We checked the proportional hazards assumption prior to performing the univariable analysis by using the Kaplan-Meier method. To avoid multicollinerality among the ambient air pollution datasets, only PM_2.5_ was included in the multivariable analysis. We used Stata (version 12) to perform all of the statistical analyses.

### Ethical approval

Ethical approval was granted by the Chiang Mai University Ethics Committee (No 200/2021) from the Faculty of Medicine. Due to anonymous data recorded in the present study, the requirements for written informed consent were waived by the Research Ethics Committee of the Faculty of Medicine at Chiang Mai University.

## Results

Of the 540 patients with hematologic malignancy, 335 (62.0%) were male, most were diagnosed at 1–10 years of age (66.7%), with leukemia being the most common diagnosis (73.9%), and 12.3% came from families with a history of smoking (S1 Table in S1 File. Of the 499 patients with solid tumors, the proportions of male and female were similar (52.9% vs. 47.1%), most of whom were diagnosed when less than 10 years old (63.3%). Cancer of the brain or nervous system was the most common diagnosis (37.08%), followed by bone (11.02%), kidney (9.22%), liver (6.41%), and eye (6.01%). The incidence of a history of smoking in the family was 13.9%. The all-cause mortality rates of the patients within 10 years after diagnosis with hematologic malignancy or solid tumors were 36.3% and 42.1%, respectively (S2 Table in S1 File).

According to the results in Table 1, 199 childhood hematologic malignancy patients died from any cause, with the overall mortality rate being 5.3 per 100 PYFU (95% CI = 4.6–6.0).

**Table 1:** Childhood hematologic malignancy and mortality rates according to the baseline characteristics.

Characteristic		Survived (n (%))	Died (n (%))	PYFU	Mortality Rate*	95%CI
**Overall**		341 (63%)	199 (37%)	3790	5.3	4.6–6.0
**Gender**						
	Male	218 (65%)	117 (35%)	2386	4.9	4.1–5.9
	Female	123 (60%)	82 (40%)	1403	5.8	4.7–7.3
**Age at diagnosis (years old)**						
	< 1	15 (47%)	17 (53%)	171	10.0	6.2–16.0
	1–10	242 (67%)	118 (33%)	1212	4.5	3.8–5.4
	≥ 10	84 (57%)	64 (43%)	1006	6.3	5.0–8.1
**Year of enrollment**						
	2003–2013	236 (59%)	167 (41%)	3275	5.1	4.4–5.9
	2014–2018	105 (77%)	32 (23%)	515	6.2	4.4–8.8

*per 100 PYFU (persons per year of follow-up); CI, confidence interval.

The mortality rate for boys was 4.9 per 100 PYFU (95% CI = 4.1–5.9) while that for girls was 5.8 per 100 PYFU (95% CI = 4.7–7.3). Age at diagnosis < 1 year old had the highest mortality rate of the age groups (10.0 per 100 PYFU; 95% CI = 6.2–16.0). Enrollment between 2014 and 2018 had a higher mortality rate than in the years beforehand (6.2 per 100 PYFU (95% CI = 4.4–8.8)).

**Fig 2:**
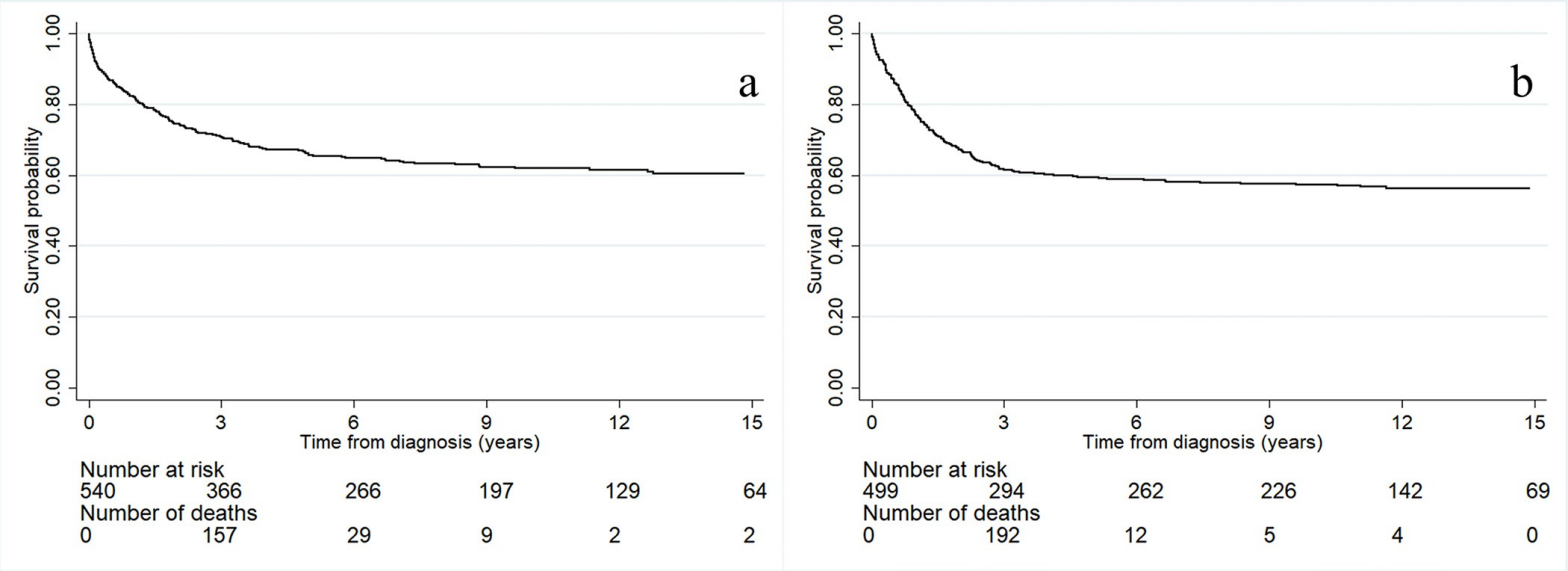
The survival rates of the childhood cancer according to (a) hematologic malignancy patients (b) solid tumor patients.

Fig 2(A) presents the overall survival rates of the childhood hematologic malignancy patients. Within the first three years after diagnosis, 157 patients died and the survival probability sharply dropped to 71%. Three years after diagnosis, the survival probability slowly decreased throughout the follow-up time; the survival rates at 6, 9, and 12 years after diagnosis were 65%, 62%, and 62%, respectively.

Fig 2(B) presents the overall survival rates of the childhood solid tumor patients. 192 patients died within the first three years of diagnosis, the survival probability sharply dropped to 61%. Three years after diagnosis, the survival probability slowly decreased throughout the follow-up period (the survival rates at 6, 9, and 12 years after diagnosis were 59%, 58%, and 56%, respectively).

**Fig 3:**
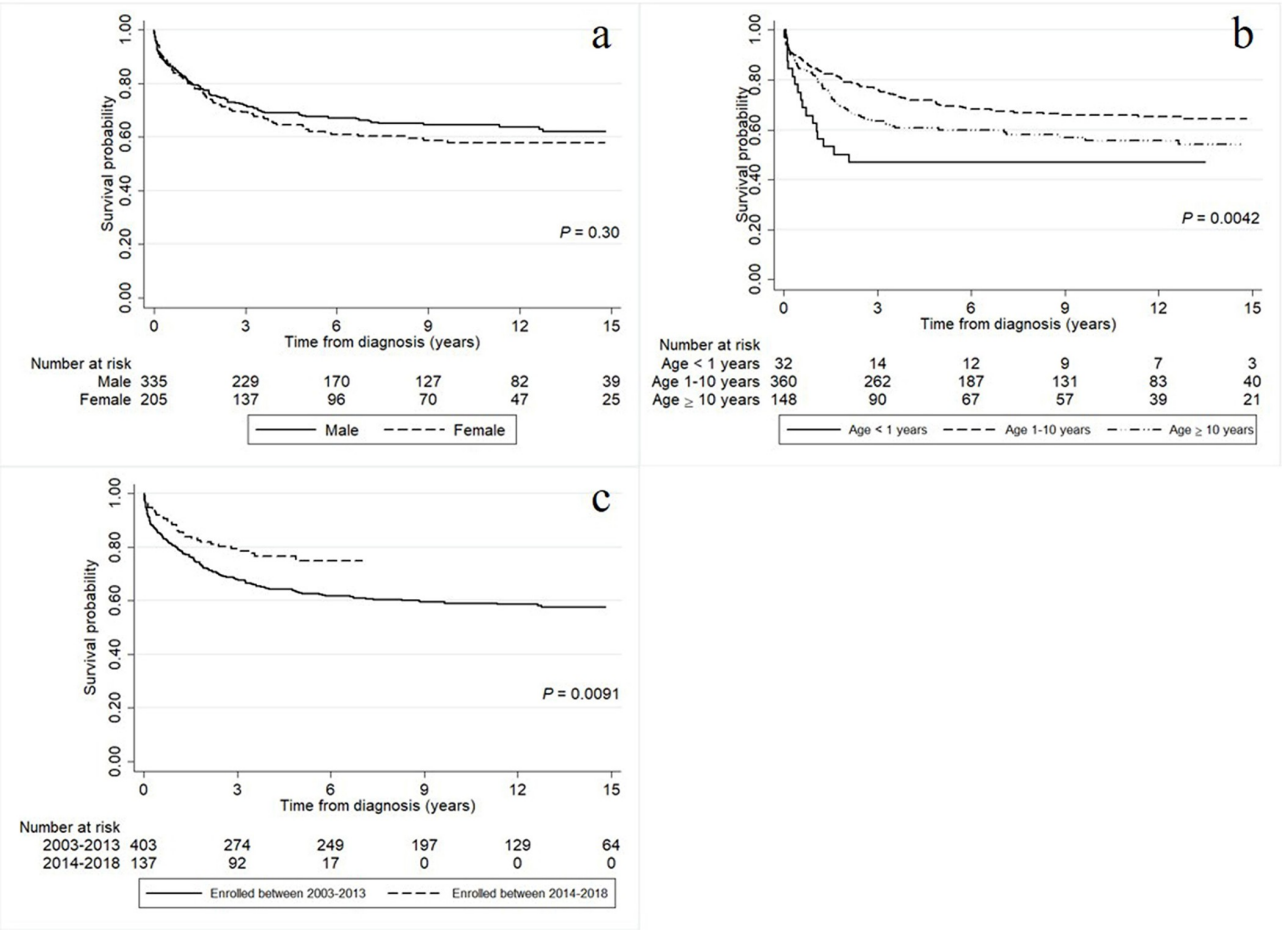
The survival rates of the childhood hematologic malignancy patients according to (a) gender, (b) age at diagnosis, and (c) year of enrollment.

The survival rates of the childhood hematologic malignancy patients according to baseline characteristics including gender, age at diagnosis, and calendar year of enrollment are presented in Fig 3. The results in Fig 3(B) suggest that patients who were < 1 or ≥ 10 years old had a significantly lower survival rate than those between these age groups. In addition, patients who enrolled between 2003 and 2013 had a significantly lower survival rate than those who enrolled between 2014 and 2018 (Fig 3(C)).

Table 2 reports the results of Cox proportional hazard models for determining risk factors associated with the risk of death among the childhood hematologic malignancy patients. The results of the univariable analysis show that age at diagnosis and calendar year of enrollment were risk factors (both *P* ≤ 0.05).

**Table 2:** Risk factors associated with death among the childhood hematologic malignancy patients.

Characteristic	Died	Total	Univariable Analysis			Multivariable Analysis		
			HR	95% CI	P*	aHR	95% CI	*P* *
**At diagnosis**								
Female	82	205	1.16	0.87–1.54	0.31	–	–	–
Age < 1 year old	17	32	2.08	1.25–3.46	0.0076	2.07	1.25–3.45	0.0078
Age ≥ 10 years old	64	148	1.41	1.04–1.91		1.41	1.04–1.91	
Enrolled between 2003 and 2013	167	403	1.65	1.13–2.42	0.0066	1.65	1.13–2.42	0.0067
**Time-updated variables**								
PM_2.5_ concentration ≥ 40 μg/m^3^	–	–	0.95	0.69–1.31	0.76	–	–	–
PM_10_ concentration ≥ 55 μg/m^3^	–	–	0.98	0.72–1.34	0.92	–	–	–
NO_2_ concentration ≥ 8.5 ppb	–	–	0.93	0.67–1.30	0.68	–	–	–
SO_2_ concentration ≥ 7.7 ppb	–	–	0.85	0.61–1.20	0.35	–	–	–
CO concentration > = 410 ppb	–	–	1.11	0.83–1.49	0.47	–	–	–
O_3_ concentration ≥ 36 ppb	–	–	1.16	0.88–1.55	0.29	–	–	–

*P-value obtained from a partial likelihood ratio test. HR, hazard ratio; CI, confidence interval; aHR, adjusted hazard ratio.

The age at diagnosis and calendar year of enrollment were included in the multivariable analysis. We found that they were independently associated with the risk of death (both *p*-values < 0.05). Childhood hematologic malignancy patients who were < 1 and ≥ 10 years old at diagnosis had a higher risk of death than those in between (adjusted hazard ratio [aHR] = 2.07; 95% CI = 1.25–3.45 and aHR = 1.41; 95% CI = 1.04–1.91, respectively). In addition, we also found that those enrolled between 2003 and 2013 had an increased risk of death (aHR = 1.65; 95% CI = 1.13–2.42).

According to the results in Table 3, 214 childhood solid tumor patients died from any cause, with the overall mortality rate being 5.9 per 100 PYFU. (95% CI = 5.1–6.7). The mortality rate of males was 6.9 per 100 PYFU (95% CI = 5.8–8.2) while that of females was 4.9 per 100 PYFU (95% CI = 4.0–6.0). Age at diagnosis < 10 years old had a higher mortality rate than ≥ 10 years old (6.0 per 100 PYFU; 95% CI = 5.0–7.1). When considering the three cancer stages, the mortality rate was highest in the metastatic stage group (10.3 per 100 PYFU; 95% CI = 8.3–12.9). Finally, the mortality rate was 13.2 per 100 PYFU (95% CI = 9.1–19.1) in patients who enrolled between 2014 and 2018.

**Table 3:** Baseline characteristics of childhood solid tumor patients and mortality rate.

Characteristic		Survived (n (%))	Died (n (%))	PYFU	Mortality Rate*	95% CI
Overall		285 (57%)	214 (43%)	3646	5.9	5.1–6.7
**Gender**						
	Male	141 (53%)	123 (47%)	1782	6.9	5.8–8.2
	Female	144 (61%)	91 (39%)	1863	4.9	4.0–6.0
**Age at diagnosis (years)**						
	<10	182 (58%)	134 (42%)	2250	6.0	5.0–7.1
	≥10	103 (56%)	80 (44%)	1396	5.7	4.6–7.1
**Cancer stage**						
	Localized	122 (69%)	54 (31%)	1578	3.4	2.6–4.5
	Regional	91 (58%)	67 (42%)	1098	6.1	4.8–7.7
	Metastatic	59 (43%)	77 (57%)	745	10.3	8.3–12.9
**Calendar year of enrollment**						
	2003–2013	243 (57%)	186 (43%)	3433	5.4	4.7–6.3
	2014–2018	42 (60%)	28 (40%)	212	13.2	9.1–19.1

*per 100 PYFU (persons per year of follow-up); CI, confidence interval.

**Fig 4:**
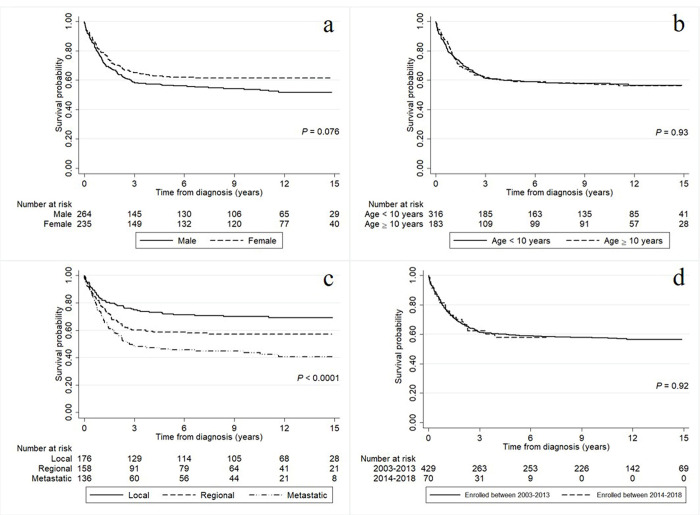
The survival rates of the childhood solid tumor patients according to gender (a), age at diagnosis (b), cancer stage (c), and calendar year of enrollment (d).

The survival rates of the childhood solid tumor patients according to baseline characteristics including gender, age at diagnosis, cancer stage, and calendar year of enrollment are presented in Fig 4. The results in Fig 4(C) suggest that those diagnosed with the metastatic stage had a significantly lower survival rate than the other two stages (both *P* < 0.0001).

Table 4 represented the results of Cox proportional hazard models for determining the risk factors associated with the risk of death among the childhood solid tumor patients. The results of the univariable analysis show that the cancer stage provided the highest risk factor (*P* < 0.0001). When including gender and the cancer stage in the multivariable analysis, we found that only the cancer stage remained in the final model, and so only univariable analysis was performed. The metastatic cancer stage (HR = 2.26; 95% CI = 1.60–3.21) and the regional cancer stage (HR = 1.53; 95% CI = 1.07–2.19) were both associated with an increased risk of death among the childhood solid tumor patients.

**Table 4:** Risk factors associated with death among the childhood solid tumor patients.

Characteristic	Died	Total	Univariable analysis		
			HR	95% CI	*P* *
**At diagnosis**					
Male	123	264	1.28	0.97–1.67	0.076
Age ≥ 10 years old	80	183	1.01	0.77–1.34	0.93
Regional cancer stage	67	158	1.53	1.07–2.19	<0.0001
Metastatic cancer stage	77	136	2.26	1.60–3.21	
Enrolled between 2003 and 2013	157	357	0.98	0.66–1.46	0.92
**Time-updated variables**					
PM_2.5_ concentration ≥ 40μg/m^3^	–	–	0.93	0.70–1.23	0.60
PM_10_ concentration ≥ 55 μg/m^3^	–	–	0.88	0.66–1.15	0.34
NO_2_ concentration ≥ 8.5 ppb	–	–	1.10	0.81–1.46	0.58
SO_2_ concentration ≥ 7.7 ppb	–	–	1.05	0.77–1.42	0.77
CO concentration ≥ 410 ppb	–	–	0.96	0.73–1.26	0.75
O_3_ concentration ≥ 36 ppb	–	–	0.99	0.75–1.32	0.97

*P obtained from a partial likelihood ratio test. HR, hazard ratio; CI, confidence interval.

## Discussion

Air pollution is a serious global concern, with several air pollutants having been classified as group 1 human carcinogens [31]. Long-term exposure to air pollutants is known to cause internal organ damage and chronic inflammation, as well as deleteriously affecting the immune system [32]. Although the mechanism for cancer development due to air pollutant exposure is not thoroughly understood, it has been shown to shorten telomeres and interfere with the DNA damage and repair process [33], which along with the influence of systemic inflammation, could be related to inflammation-related carcinogenesis [34, 35]. Hoffmann et al. [36] reported that a 3.91 μg/m^3^ increase in PM_2.5_ was associated with increased inflammatory markers. Moreover, air pollution exposure negatively impacts cancer mortality [37].

Although the relationship between adult cancer mortality and specific air pollutant exposure, including PM_2.5_ [17–19, 21–23, 37–42], PM_10_ [19, 21–23, 43–46], NO_2_ [19, 20, 22, 23, 47, 48], SO_2_ [20, 47–50], CO [20], and O_3_ [20, 51], has been extensively investigated, the association between air pollution and the survival rate of children with cancer has not.

Since air pollution has become a significant concern in northern Thailand, we investigated whether exposure to it is the mortality risk factor for children with hematologic malignancy or solid tumors in eight provinces in upper northern Thailand. We found that residential concentrations of PM_2.5_, PM_10_, NO_2_, SO_2_, CO, and O_3_, were not significantly associated with the risk of death in pediatric patients with these cancers. Similarly, no significant association between all-cause mortality and various cancer types and PM_2.5_ exposure was found in a study involving 2,444 pediatric patients in Utah [37]. This could be because the effects of aging, exposure to environmental mutagens, and the accumulation of mutations over time contribute to cancer development [52]. Moreover, the positive relationship between air pollution and mortality in older patients with cancer could be inflammation-related [34, 35]. We assume that the shorter duration of air pollution exposure in children with cancer reduces the impact of the former on their mortality.

However, deeper analysis of the Utah Cancer Registry revealed a significant association between PM exposure and mortality when classified by specific cancer types [37]. The authors found that both cancer mortality and all-cause mortality in children with lymphoma or CNS tumors at 5 and 10 years postdiagnosis, respectively, were significantly associated with a PM_2.5_ concentration of 5 μg/m^3^. They also revealed the significant association of a PM_2.5_ concentration of 5 μg/m^3^ with the all-cause mortality of children with lymphoid leukemia and hepatic tumors at 5 and 10 years postdiagnosis, respectively. Moreover, PM_2.5_ ≥ 12 μg/m^3^ was positively associated with all-cause mortality of pediatric lymphoid leukemia patients. However, we could not perform statistical analysis based on specific cancer types due to the limited sample size in our study. Therefore, whether air pollution exposure affects the mortality of children with specific cancer types was not investigated.

Apart from air pollution, we found a significant association between the age at diagnosis and mortality. Pediatric hematologic cancer patients < 1 or ≥ 10 years old at diagnosis posed a significant risk factor of death compared to 1–10 years old (*P* = 0.0078). This finding is similar to those from previous studies [53–55], which can be explained by the risk classification of pediatric leukemia, especially acute lymphoblastic leukemia and acute myeloid leukemia, both of which are common childhood hematologic malignancies; a greater risk at < 1 year old than 1–10 years old due to poorer genetic and molecular development in the former age group [56, 57].

Nevertheless, age at diagnosis was not a significant risk factor for the solid pediatric tumor group. According to a report by the Italian Association of Paediatric Haematology and Oncology [53], superior survival times of patients with neuroblastoma or Wilms’ tumor were observed at < 1 year old, which is a similar finding to that from a study in Sweden [58]. Meanwhile, patients aged 0–4 years old with Ewing sarcoma or intracranial primitive neuroectodermal tumor had poor survival times. Children with rhabdomyosarcoma or embryonal sarcoma had better survival durations when diagnosed at 5–9 years old than at < 1 or 10–14 years old [53, 58]. Similarly, patients aged 10–14 years old with Wilms’ tumor or astrocytoma had poor survival times compared to those aged < 10 years old [53]. Therefore, the diversity of solid tumors and the differences in prognosis and survival times among the different age groups could be a reason for the findings in our study.

We found a significant improvement in survival times in children with hematologic malignancy when enrolled during 2014–2018 compared to 2003–2013 (*P* = 0.0091), which is similar to the findings from previous studies [53, 55, 59]. The evolution of knowledge about and treatment strategies for hematologic malignancy could be the reason for this improvement. In Thailand, the Thai Pediatric Oncology Group (ThaiPOG) was established in 2002 to develop a pediatric cancer registry and a national standard chemotherapy protocol with the goal of more efficacious treatment and outcomes. The ThaiPOG launched the first national leukemia and lymphoma treatment protocols in 2006, while protocols for other childhood cancers were established in 2014. These in conjunction with better supportive care for pediatric cancer patients have led to improved survival times. However, this is not the case for solid tumors (*P* = 0.92). The population-based EUROCARE-5 project [55] running from 1999–2007 reported no significant difference in survival times among children with various solid tumors. Similarly, in a single-center study in Finland comparing the periods 1990–1999 and 2000–2015 [60], the authors reported no significant change in survival times even with the improvements in diagnosis and treatment. Contrarily, a report from the Swedish Childhood Solid Tumour Working Group [58] and the Texas Cancer Registry [61] showed significant improvements in survival times. Other factors such as cancer stage [60, 61], the prognostic molecular and genetic basis of cancer [62, 63], the availability of treatment modalities, and response to treatment can impact the survival outcomes of children with solid tumors. Patients with advanced-stage cancer usually have adverse outcomes. Similarly, we found a more significant association between advanced-stage cancer and mortality compared to the localized cancer stage group.

This present study is the first to enabled PM_2.5_, PM_10_, NO_2_, SO_2_, CO, and O_3_ levels over the past 15 years as a time-varying covariate in the Cox proportional hazard model for the evaluation of risk factors of death among childhood cancer patients. There were some limitations of our study. First, we analyzed the association of air pollution with all-cause mortality rather than for specific cancers. Second, we could not analyze specific cancer types and the differences in treatment modality and prognosis due to the limited sample size. Third, we assumed a low proportion of relocation of the participants and did not account for their possible relocation during the study period, which could have caused location-based bias in our findings. Finally, we could not conduct subgroup analysis by area due to the limited number of patients. Further study with a larger sample size should be conducted to investigate differences in the effect of air pollution on specific cancer types both for the whole of the study area and the specific areas mentioned earlier. Therefore, air pollution exposure should be avoided in vulnerable childhood cancer patients to prevent further tissue damage and inflammation. Furthermore, the prospective large-scale studies may be required to identify the powerful causal relationship of environmental exposure with mortality in childhood cancer.

## Conclusions

We found the significant association of mortality with the age at diagnosis and calendar year of enrollment in hematologic malignancy, and the association of mortality with the stage at diagnosis in solid tumors. These findings based on the character of risk classification, the development of treatment modality over time and the inferior outcome in metastatic group. We could not identify the association between air pollution exposure and all-cause mortality in pediatric patients with both hematologic malignancy and solid tumors. Further large-scale analytical study is required to elucidate the possible relationship of air pollution and mortality in childhood cancer.

## Supporting information

S1 File (DOCX)
