# Supplementary material for: Ambient air pollution as a time-varying covariate in the survival probability of childhood cancer patients in the upper Northern Thailand
Source: PLoS One. 2024 May 10;19(5):e0303182. doi: 10.1371/journal.pone.0303182 (PMC11086912; doi:10.1371/journal.pone.0303182)
Supplement: S1 File — (DOCX) [file pone.0303182.s001.docx]

**S1 Table.** **Demographics of the patients with hematologic malignancy (*N* = 540).**

| **Characteristic** | **n** | **%** |
| --- | --- | --- |
| **Gender** |  |  |
| Male | 335 | 62.0 |
| Female | 205 | 38.0 |
| **Age at diagnosis (years old)** |  |  |
| < 1 | 32 | 5.9 |
| 1–10 | 360 | 66.7 |
| ≥10 | 148 | 27.4 |
| **A family history of smoking** | **54** | **12.3** |
| **Diagnosis year** |  |  |
| 2003–2013 | 403 | 74.6 |
| 2014–2018 | 137 | 25.4 |
| **Type of cancer** |  |  |
| Leukemia | 399 | 73.9 |
| Lymphoma | 137 | 25.4 |
| Myelodysplastic/myeloproliferative neoplasm | 4 | 0.7 |
| **Number of all-cause deaths within 10 years** | **196** | **36.3** |

**S2 Table. Demographics of the patients with solid tumors (*N* = 499).**

| **Characteristic** | ***n*** | **%** |
| --- | --- | --- |
| **Gender** |  |  |
| Male | 264 | 52.9 |
| Female | 235 | 47.1 |
| **Age at diagnosis** |  |  |
| <10 | 316 | 63.3 |
| ≥10 | 183 | 36.7 |
| **A family history of smoking** | **43** | **13.9** |
| **Diagnosis year** |  |  |
| 2003–2013 | 429 | 86.0 |
| 2014–2018 | 70 | 14.0 |
| **Type of cancer** |  |  |
| Adrenal gland | 14 | 2.81 |
| Bladder | 3 | 0.6 |
| Bone | 55 | 11.02 |
| Brain, nervous system | 189 | 37.88 |
| Colon | 1 | 0.2 |
| Connective and soft tissue | 24 | 4.81 |
| Eye | 30 | 6.01 |
| Immunoproliferative diseases | 2 | 0.4 |
| Kaposi sarcoma | 1 | 0.2 |
| Kidney | 46 | 9.22 |
| Liver | 32 | 6.41 |
| Malignant melanoma | 3 | 0.6 |
| Mouth | 1 | 0.2 |
| Nasopharynx | 9 | 1.8 |
| Nose, sinuses, etc. | 3 | 0.6 |
| Other and unspecified | 13 | 2.61 |
| Other endocrine | 15 | 3.01 |
| Other male genital organs | 1 | 0.2 |
| Other skin | 4 | 0.8 |
| Other thoracic organs | 7 | 1.4 |
| Ovary | 12 | 2.4 |
| Pancreas | 1 | 0.2 |
| Placenta | 1 | 0.2 |
| Salivary glands | 3 | 0.6 |
| Small intestine | 1 | 0.2 |
| Stomach | 1 | 0.2 |
| Testes | 5 | 1 |
| Thyroid | 18 | 3.61 |
| Tongue | 1 | 0.2 |
| Trachea, bronchus, and lung | 2 | 0.4 |
| Vagina | 1 | 0.2 |
| **Stage at diagnosis** |  |  |
| Localized | 176 | 37.5 |
| Regional | 158 | 33.6 |
| Metastatic | 136 | 28.9 |
| **Number of all-cause deaths within 10 years** | **210** | **42.1** |
